# KDM5C and KDM5D influence DNA methylation in adult mouse liver

**DOI:** 10.1186/s13293-026-00844-6

**Published:** 2026-02-12

**Authors:** Emily Gibbons, Kathleen Oros Klein, Shinya Inoue, Tohru Kimura, Celia M. T. Greenwood, Anna K. Naumova

**Affiliations:** 1 Department of Human Genetics, McGill University, Montréal, Québec Canada; 2 Lady Davis Institute for Medical Research, Montreal, Québec Canada; 3 Laboratory of Stem Cell Biology, Department of Biosciences, Kitasato University School of Science, 1-15-1 Kitasato, Minami-ku, Sagamihara, Kanagawa Japan; 4 Gerald Bronfman Department of Oncology, Department of Epidemiology, Biostatistics & Occupational Health, McGill University, Montréal, Québec Canada; 5 The Research Institute of the McGill University Health Centre, Montréal, Québec Canada; 6 Department of Obstetrics and Gynecology, McGill University, Montréal, Québec Canada

**Keywords:** DNA methylation, Mouse, Sex chromosome

## Abstract

**Background:**

Several lines of evidence suggest that the sex-chromosome complement influences autosomal gene regulation and DNA methylation, however, the exact molecular mechanisms responsible for such effects remain elusive. X-linked epigenetic modifiers that escape X-chromosome inactivation, and hence have higher dosage in female cells, are the primary gene candidates for mediating the effects of X-dosage, whereas Y-linked paralogs may rescue such imbalance or have distinct effects on methylation.

**Methods:**

Here, we tested the impacts of mutations in mouse histone lysine 4 demethylases *Kdm5c* (X-linked) and *Kdm5d* (Y-linked) on DNA methylation in mouse liver. KDM5C and KDM5D demethylate H3K4me2/3 thereby facilitating DNA methylation of their target DNA regions. Therefore, loss of either *Kdm5c* or *Kdm5d* is expected to reduce DNA methylation at such regions. We hypothesized that *Kdm5c* gene dosage was responsible for the X-dosage dependent DNA methylation in mouse liver and compared DNA methylation patterns in heterozygous mutant *Kdm5c+/-* and wild type females using whole genome bisulfite sequencing (WGBS) and DSS.

**Results:**

We examined the impacts of mutations in *Kdm5c* or *Kdm5d* on genome-wide DNA methylation and found that they had different targets but tended to map close to H3K4me1-enriched regions. We also compared the *Kdm5c* and *Kdm5d* sensitive regions to regions with sex-chromosome complement dependent DNA methylation and found no overlaps.

**Conclusions:**

In summary, while *Kdm5c* and *Kdm5d* have multi-locus effects on DNA methylation in mouse liver, they are unlikely to be solely responsible for sex-chromosome complement effects on DNA methylation in adult mouse liver.

**Supplementary Information:**

The online version contains supplementary material available at 10.1186/s13293-026-00844-6.

## Plain Language summary

Several lines of evidence suggest that sex chromosomes influence gene regulation across the genome, however, the exact molecular mechanisms of such influence remain unknown. Certain genes on the X chromosome escape X-chromosome inactivation, and hence have higher dosage in female cells with two X chromosomes compared to male cells with one X and one Y. These are the primary gene candidates for mediating the effects of the different numbers of X chromosomes in males and females. Y-linked counterparts of such genes may rescue the imbalance or have separate effects on gene regulation. DNA methylation plays a critical role in gene regulation and is often used as a measure of gene activity. Here, we examined how mutations in mouse histone lysine 4 demethylases *Kdm5c* (X-linked) and *Kdm5d* (Y-linked) influenced DNA methylation in mouse liver and tested the hypothesis that higher *Kdm5c* gene dosage in females was responsible for the X-chromosome dosage dependent DNA methylation in mouse liver. We examined the impacts of mutations in *Kdm5c* or *Kdm5d* on genome-wide DNA methylation and found that they had different targets but tended to map close to regulatory regions with predominantly enhancer-type epigenetic features. We also compared the *Kdm5c* and *Kdm5d* sensitive regions to regions with sex-chromosome complement dependent DNA methylation and found no overlaps. In summary, while *Kdm5c* and *Kdm5d* have multi-locus effects on DNA methylation in mouse liver, they are unlikely to be solely responsible for sex-chromosome complement effects on DNA methylation in adult mouse liver.

**Supplementary Information:**

The online version contains supplementary material available at 10.1186/s13293-026-00844-6.

## Highlights

We tested the impacts of mutations in sex-chromosome-linked genes encoding histone H3K4 demethylases, *Kdm5c* and *Kdm5d*, on sex-biased DNA methylation in adult mouse liver and determined that:

- Mutations in *Kdm5c* and *Kdm5d* induce significant changes in DNA methylation in adult mouse liver, primarily at autosomal loci;
- Functional annotation indicates that loss of *Kdm5c* affects DNA methylation at promoter and enhancer regions enriched for histone H3K4me3 or H3K4me1 and H3K27ac marks, whereas loss of *Kdm5d* induces changes in methylation near enhancer regions;
- Overall, our data show that KDM5C and KDM5D are not the primary mediators of sex-chromosome complement effects on DNA methylation in the adult liver. However, interaction with other factors cannot be ruled out. These results are novel and add to the understanding of mechanisms responsible for sex-bias in the epigenome.

**Supplementary Information:**

The online version contains supplementary material available at 10.1186/s13293-026-00844-6.

## Background

Sex differences in mammalian gene regulation have been reported across different tissues and developmental stages. DNA methylation is a highly conserved molecular mechanism implicated in genome regulation and stability across different species with a well-documented function in gene silencing (reviewed in [1, 2]). In agreement with this function is the observation that sex-biased DNA methylation levels largely mirror sex-biased gene expression, however the exact roles of different factors in the establishment and maintenance of sexual dimorphism in DNA methylation remain elusive [3–7]. To date, the mouse liver has been one of the best studied organs with respect to sexual dimorphism in gene regulation due to its relative homogeneity (about 80% of cells are hepatocytes) and the largest number of sex-biased genes compared to other adult non-reproductive organs, apart from kidney [5, 8–10]. In principle, sex bias in DNA methylation may be driven by the gonadal sex hormone-associated pathways or the sex-chromosome complement [11–14]. Gonadal sex hormones and the pituitary signalling pathways explain the majority of sex differences in liver DNA methylation [3, 5, 8]. However, our recent work demonstrated that the sex-chromosome complement also influenced autosomal DNA methylation in adult mouse liver [3]. More specifically, we identified sex-chromosome complement dependent autosomal differentially methylated regions (DMRs) in pair-wise comparisons between female mice with different sex-chromosome complements, e.g. XX and sex-reversed XY females and XX vs. XO females [3]. Moreover, data from several studies suggest that the onset of sex-biased transcription and/or DNA methylation at certain loci, including genes for critical hepatic transcription factors such as CUX2, ONECUT1, FOXA1, and FOXA2, takes place before puberty, prompting the question about the involvement of the sex chromosomes [5, 15, 16]. Mechanisms underlying the impact of the sex-chromosome complement on DNA methylation remain elusive. One of the possible scenarios is that the influence of X-chromosome dosage on methylation is mediated by X-linked epigenetic modifiers that escape X-chromosome inactivation [11, 12]. In this context, one X-Y gene pair is of particular interest: *Kdm5c* and *Kdm5d* that encode histone lysine 4 demethylases. Both catalyze demethylation of H3K4me2/3 but not H3K4me1. Moreover, *KDM5C/Kdm5c* escapes X-inactivation in both humans and mice and has higher expression in female than male somatic cells [17, 18].

In humans, mutations in *KDM5C* cause intellectual disability [19] and *KDM5C* dosage is associated with sex-biased DNA methylation at autosomal loci [20, 21]. In mice, induced mutations in *Kdm5c* and *Kdm5d* impact a range of phenotypes from developmental to behavioural traits and demonstrate the functional importance of these genes for mouse biology [22–24]. Homozygous female mice that lack functional KDM5C die either early during embryonic development or at birth, with severity of the phenotype depending on the type of mutation and genetic background [22, 23, 25]. Hemizygous males survive due to the presence of *Kdm5d*, whereas males lacking both *Kdm5c* and *Kdm5d* are small and die at birth, i.e. show the same phenotypes as homozygous *Kdm5c* mutant females [22]. Heterozygous mutant females are viable and fertile but display altered memory and behaviour [22, 24]. Moreover, heterozygosity for a *Kdm5c* mutation in females recapitulates some of the sexual dimorphism seen in adipocyte biology, implicating KDM5C as a mediator of X-chromosome dosage effects in adipocytes [23]. In male ESCs, i.e. before sex determination and formation of fetal gonads, a *Kdm5c* loss-of-function mutation results in a sex-reversed expression pattern [26]. It is also important to note that in these mutant mice, the presence of two other functional H3K4 demethylases, KDM5A (chr 6) and KDM5B (chr 1) does not rescue the adverse phenotypes, suggesting that the functions of different members of the KDM5 family are not redundant.

Thus, the ensemble of current data justifies the study of the pair KDM5C/KDM5D as good candidates that may explain some of the effects of the sex-chromosome complement on global DNA methylation. Therefore, we investigated the influence of *Kdm5c* and *Kdm5d* mutations on genome-wide DNA methylation patterns in adult mouse liver and tested the hypothesis that these genes contributed to sex-biased DNA methylation.

## Materials and methods

### Mouse crosses

**Fig. 1:**
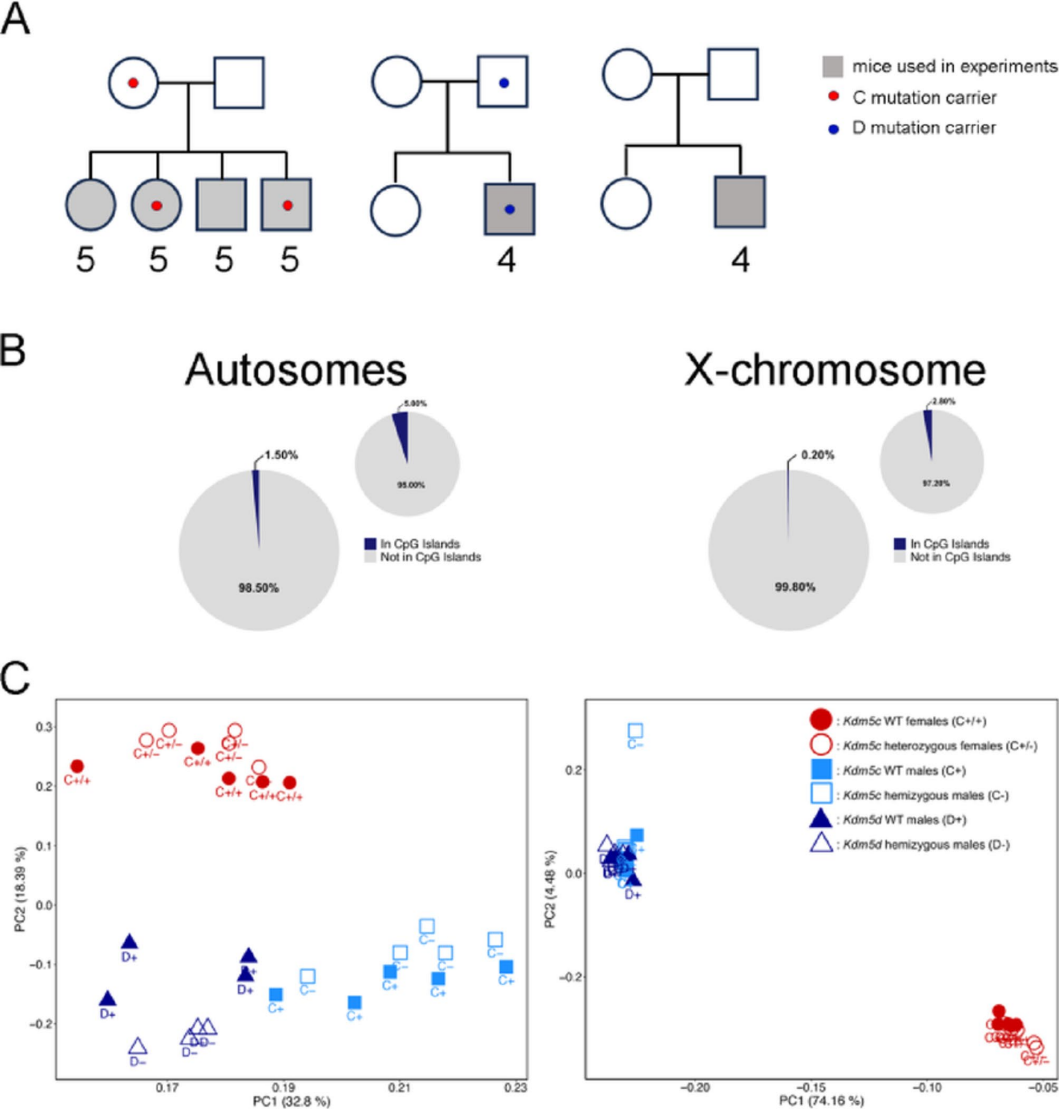
DNA methylation is influenced by sex and genetic background. (**A**) Mouse crosses used to generate mutant mice and controls. Filled grey shapes indicate mice whose DNA was used for WGBS. Number of mice is indicated below each symbol. (**B**) Representation of the proportion of CpG islands remaining in the dataset after filtering for autosomal CpGs (left) and X chromosomal CpGs (right) in comparison to proportions before filtering. (**C**) Principal component analysis (PCA) of DNA methylation at the 2500 most variable autosomal (left) and X-chromosomal (right) CpGs

Mice carrying a 172-bp deletion in the X-linked gene *Kdm5c* (referred to from this point on as C- for males or C+/- for heterozygous females) or a 2-bp deletion in the Y-linked gene *Kdm5d* (referred to as D- from this point on) were described previously [22]. Both mutations were maintained on an ICR strain background (Fig. 1 A). Genomic DNA from livers of 10–11 week-old mutant mice and their wild type littermates or control crosses were extracted using a standard proteinase K, phenol/chloroform procedure. All animal care and experimental procedures were performed in accordance with the Guidelines of Animal Experiments of Kitasato University and were approved by the Institutional Animal Use and Care Committee of Kitasato University.

### Whole genome bisulfite sequencing (WGBS) and data preprocessing

A total of 28 liver DNA samples were sequenced: 5 wild type females (C+/+), 5 WT males (C+), 5 C+/- females, and 5 C- males from the C-cross, 4 D- males and 4 wild type males (D+) from the D cross and wild type control (Fig. 1 A). Whole genome bisulfite sequencing (WGBS) was performed at the McGill Genome Centre using paired-end sequencing. Raw data were processed with the GenPipes MethylSeq pipeline (v3.6.2; [27]), which includes read trimming with Trimmomatic and alignment to the mm10 genome (GRCm38) using Bismark. CpG methylation calls were generated with Bismark Methylation Extractor.

### Data filtering

To minimize the impact of genetic variation on methylation measurements, all CpG sites overlapping annotated single nucleotide polymorphisms (SNPs) in the Mouse Genome Project were excluded. CpGs overlapping with SNPs were identified and removed using the *findOverlaps*() function from the GenomicRanges package ([v 1.61.1] [28]),. In addition, regions listed in the mm10 ENCODE Blacklist, which represent genomic regions prone to artifacts, were removed prior to downstream analyses [29].

### Detection of differentially methylated CpG (DMCs) and differentially methylated regions (DMRs) by contrasts

Differentially methylated CpG (DMCs) and regions (DMRs) were identified using DSS (v2.56.0; [30]), which models methylation differences with a Beta-Binomial distribution *via* the *DMLtest* function. DMCs were called with *callDML* (*p* < 0.05) and filtered to include only those with ≥ 10% methylation difference. DMRs were defined using *callDMR* by requiring more than two CpGs per region, with at least 50% of CpGs in the region meeting the *p* < 0.05 threshold. Adjacent DMRs within 10,000 bp were merged, and only regions with ≥ 10% methylation differences were retained. Permutation analyses confirmed the DSS results.

### Characterization of autosomal DMCs

DMCs were annotated using the *annotatr* package (v 1.35.1; [31]) with genomic features from the mm10 genome assembly, including sequence (e.g. CpG islands (CGI), shores, shelves) and genic region features (e.g. promoters, exons, introns, UTRs, coding sequences (CDS), and intergenic regions). Chromatin state annotations were derived from ENCODE mouse liver data (mixed sex, 0 days postnatal), which defines 15 chromatin states including promoter-like, enhancer-like regions ([32]; ENCODE dataset ENCSR012NQA). Chi-squared tests were used to compare the annotation distributions between DMCs from all contrasts and all other CpGs. Pairwise comparisons were conducted using Fisher’s exact tests with Bonferroni correction, and significance was defined as adjusted p-values < 0.05.

### Histone mark colocalization analysis

ENCODE ChIP-seq datasets from 8-week-old adult male mouse liver were used for histone mark colocalization analysis. ChIP-seq data for H3K4me1 (ENCSR000CAO), H3K4me3 (ENCSR000CAP), H3K27me3 (ENCSR000CEN), and H3K27ac (ENCSR000CDH) (https://www.ncbi.nlm.nih.gov/geo/query/acc.cgi?acc=GSE31039) [33] were downloaded from the ENCODE portal (mm9 genome build) and converted to the mm10 genome assembly using UCSC LiftOver. The genomic coordinates of histone peaks were then used to identify the nearest peak to each DMC identified in male mutant contrasts (C- vs. C + and D- vs. D+). Nearest peak annotation was performed using the *distanceToNearest*() function from the GenomicRanges R package ([v 1.61.1] [28]),. For each histone mark, 100 permutations for the distribution of distances to nearest peaks were generated by randomly sampling CpGs from all sites captured by the sequencing (matched in number to the observed DMCs for each contrast) and then performing nearest peak annotation for each sampled set.

### Intersecting DMRs with published datasets

To identify common targets of different *Kdm5c* mutations, we compared our C- vs. C+ DMRs with regions detected in XY *Kdm5c* knockout vs. wild-type differentiated epiblast-like cells (EpiLCs) comparison [34]. The EpiLC dataset was filtered to retain regions with q-value < 0.05. Overlaps were defined as loci located within 3 kb of each other using the *GenomicRanges* R package ([v 1.61.1] [28]),.

To evaluate overlaps between sex-chromosome dependent and *Kdm5c* or *Kdm5d*-mutation sensitive DMRs, DMRs from GSE217666 [3, 35] comparing XX.F vs. XY.F and XX^*Paf*^.F vs. XO.F were overlapped with our mutant contrasts. An overlap was counted if at least one nucleotide was shared between regions. All overlap analyses were performed using the *GenomicRanges* R package (v1.61.1) [28].

## Results

### Impact of genetic variation on genome-wide DNA methylation patterns. Filtering and quality control of DNA methylation data

Following alignment and preprocessing, we assessed CpG coverage to evaluate overall data quality and to determine appropriate filtering thresholds. Descriptive statistics calculated prior to filtering indicated that the average read depth per CpG site across samples was ~ 15x. Based on the max read count distributions, filters were applied to remove cytosines with excessively high read counts (total read counts > 100, or with counts > 40 in more than five samples). CpG sites with 0% or 100% methylation across all samples were also removed prior to differential methylation analysis.

Of the initial 22.3 million CpG sites, approximately 6.5 million were excluded due to overlap with annotated SNPs. To further reduce the potential impact of unannotated genetic variants, we excluded CpG sites that had zero coverage in any sample, as such sites may represent mismatches due to genetic variation. Next, methylated and unmethylated reads from both DNA strands were summed per CpG, and sites were retained only if strand-specific methylation proportions differed by no more than 50%. After applying all quality control and filtering steps, approximately 5.4 million CpGs remained for downstream analysis. Filtering reduced CpG island representation from 5% to 1.50% on autosomes and from 2.80% to 0.20% on the X chromosome (Fig. 1B), with the requirement for non-zero coverage having the greatest impact on this reduction.

Principal component analysis (PCA) on the 2,500 most variable CpGs showed clear separation of samples by sex and cross on both the autosomes and X chromosome. Separation by sex is consistent with prior reports of sex-biased methylation in mouse liver [3]. Among male samples, variability between the two wild-type groups (C + and D+) is likely to reflect genetic background effects, as they clustered by cross rather than forming a unified group (Fig. 1 C).

### Mutations in *Kdm5c* or *Kdm5d* are associated with both loss and gain of methylation on autosomes

Next, we identified DMCs in 6 contrasts: C+/- vs. C+/+ (DMCs sensitive to *Kdm5c* dosage in female mice); C- vs. C+ (DMCs sensitive to the *Kdm5c* mutation); D- vs. D+ (DMCs sensitive to the *Kdm5d* mutation); C + vs. D+ (DMCs sensitive to genetic background), and sex-biased DMCs obtained by contrasting wild type (C+/+ vs. C+) and mutant (C+/- vs. C-) females and males (Supplementary Tables 1 and 2). The substantial number of DMCs from the C + vs. D+ male contrast suggests that the SNP filtering was insufficient to eliminate genetic background effects. To minimize the likelihood of detecting the genetic background effects on methylation rather than the impact of the mutations, all DMCs that overlapped with those identified in the C + vs. D+ contrast were excluded from downstream analyses (Table 1, Supplementary Fig. 1, Supplementary Tables 1 and 2). The largest numbers of DMCs were found in the contrasts comparing males and females.

To examine the distribution of sex-biased DNA methylation across the X chromosome, sDMCs were binned into 2 Mb windows (Supplementary Fig. 2). In both the wild-type (C+/+ vs. C+; Supplementary Fig. 2 A), and the mutant (C+/- vs. C-; Supplementary Fig. 2B) comparisons, we observed widespread sex-bias in DNA methylation across the X chromosome. Although sDMCs that were hypermethylated in males were more numerous, this skew is likely attributable to the reduced representation of CGIs in our data. The overall distribution of sDMCs in the mutant contrast closely resembled that of the wild type.

Contrasts comparing the effects of the mutations on methylation in male livers detected comparable numbers of DMCs. In females, the *Kdm5c* mutation affected fewer CpG sites than in males. DMCs were distributed across chromosomes with both hypo- and hypermethylated CpGs detected on each chromosome (Supplementary Fig. 1 and Supplementary Table 2).

**Table 1:** Number of DMCs (*p* < 0.05) and DMRs (≥ 50% of CpGs with *p* < 0.05) identified by DSS after filtering out overlaps with the WT male contrast (C + vs. D+)

Contrast	Autosomes		X chromosome		Y chromosome	
	DMCs (hypo vs. hypermethylated)	DMRs (hypo vs. hypermethylated)	DMCs (hypo vs. hypermethylated)	DMRs (hypo vs. hypermethylated)	DMCs (hypo vs. hypermethylated)	DMRs (hypo vs. hypermethylated)
C+/- vs. C+/+	1150 (289 vs. 861)	35 (9 vs. 26)	25 (6 vs. 19)	0	-	-
C- vs. C+	1999 (483 vs. 1516)	93 (36 vs. 57)	57 (12 vs. 45)	2 (1 vs. 1)	4 (1 vs. 3)	0
D- vs. D+	2208 (1159 vs. 1049)	89 (59 vs. 30)	58 (24 vs. 34)	0	5 (1 vs. 4)	0
C+/+ vs. C+	3470 (743 vs. 2727)	184 (35 vs. 149)	3298 (2270 vs. 1028)	513 (355 vs. 158)	-	-
C+/- vs. C-	3811 (961 vs. 2850)	185 (23 vs. 162)	3268 (2331 vs. 937)	459 (317 vs. 142)	-	-

**Fig. 2:**
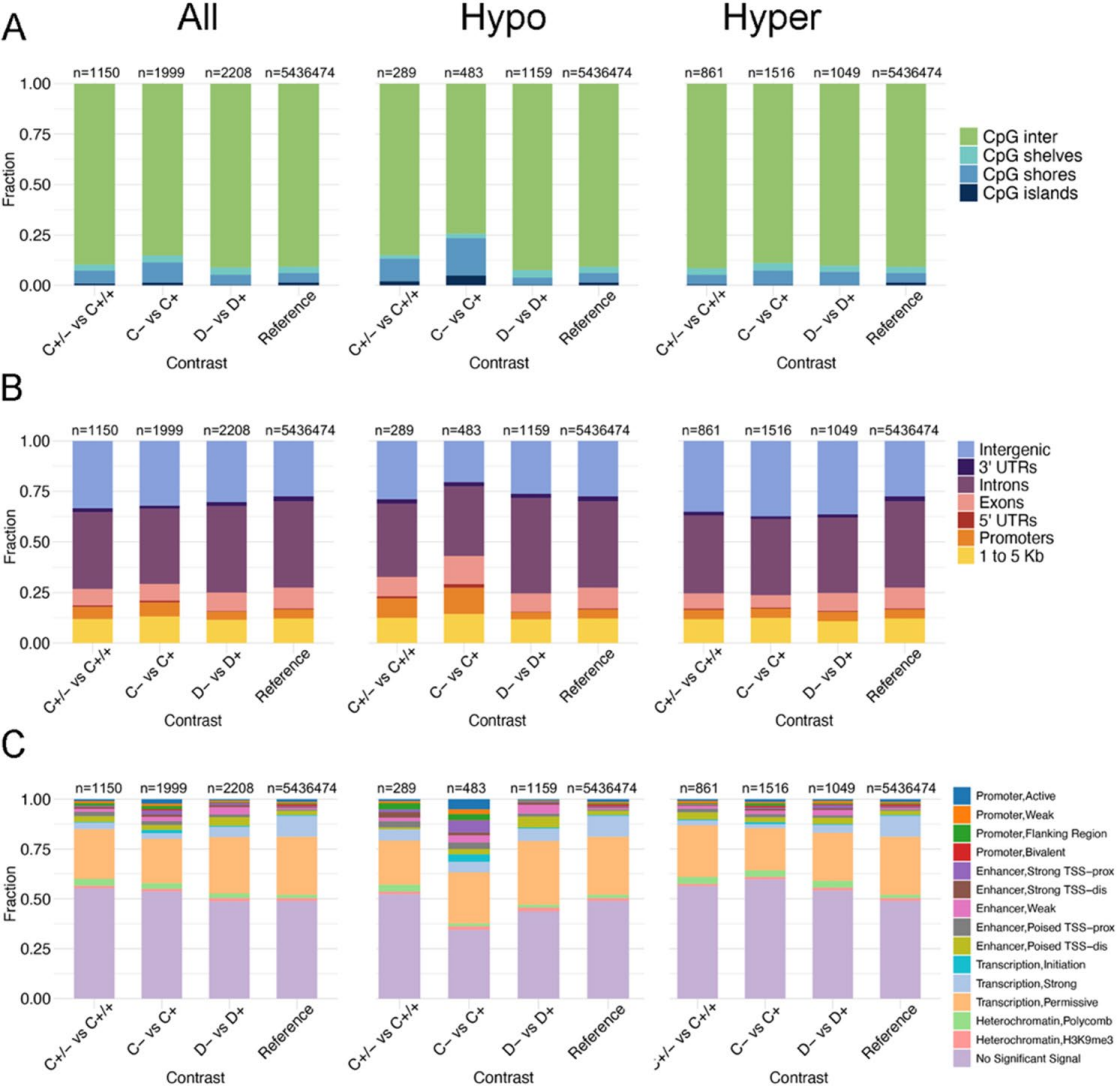
Annotation of autosomal DMCs. The y-axis represents the fraction of DMCs that overlap with each annotation type. (**A**) Sequence features annotation: “inter” refers to “open sea”. (**B**) Genic feature annotation. (**C**) Chromatin state annotation based on ChromHMM predictions from P0 mouse liver [32]. In each panel, “Reference” represents the background distribution of all tested autosomal CpGs. The number of DMCs that was annotated in each category is indicated above each bar

Next, DMCs were functionally annotated based on sequence context, genic location, and co-localization with 15 chromatin signatures, and enrichment patterns were compared by contrast (Fig. 2). For the chromatin state annotations, we used ENCODE data from neonatal liver as the first step, as P0 is currently the latest developmental stage with all 8 chromatin marks available in the ENCODE database [32].

Loss of function of the KDM5C protein in C-mutant males was associated with lower methylation of CGI shores (Fig. 2A), gene promoters (Fig. 2B) and regions with mostly active chromatin marks (Fig. 2C). Chromatin state annotation of DMCs using the ChromHMM data for neonatal liver [32] showed that regions with lower methylation in C- mutant livers (referred to as hypomethylated DMCs from this point on) were significantly enriched at active promoters (OR = 3.90, *p* = 2.83 × 10^− 6^), flanking promoter regions (OR = 6.42, *p* = 3.14 × 10^− 6^), strong enhancer regions (OR = 14.38, *p* = 1.24 × 10^− 20^) and regions of transcription initiation (OR = 8.87, *p* = 9.50 × 10^− 10^) (Fig. 2C). All the above enriched chromatin states share a common mark, the H3K4me1. Hypomethylated DMCs were additionally enriched in CpG islands (OR = 3.39, *p* = 1.54 × 10^− 5^), whereas DMCs with higher methylation in mutant livers (referred to as hypermethylated) were depleted in CpG islands (OR = 0.17, *p* = 6.31 × 10^− 5^) but modestly enriched in CpG shores (OR = 1.57, *p* = 4.32 × 10^− 4^) (Fig. 2A). Both hypo- and hypermethylated DMCs were underrepresented in regions associated with strong transcription (OR = 0.47, *p* = 0.005; OR = 0.17, *p* = 7.33 × 10^− 39^, respectively). These observations are largely consistent with the earlier reported functions of KDM5C at both promoters and enhancers [36].

Genomic annotation of *Kdm5c* dosage-sensitive DMCs (C+/- vs. C+/+) revealed no significant enrichment for specific genomic features, such as CpG islands, unlike the pronounced enrichment observed in DMCs from C- mutant males (Fig. 2A). However, hypomethylated DMCs in C+/- mutants were significantly enriched in promoter regions (OR = 2.25, *p* = 8.48 × 10^− 4^), although to a lesser extent than in C- mutant males (OR = 3.14, *p* = 6.17 × 10^− 17^). Similarly, CpG shore hypomethylation was more pronounced in male C- mutants (OR = 4.68, *p* = 2.98 × 10^− 28^) than in C+/- females (OR = 2.59, *p* = 1.06 × 10^− 04^), suggesting that CpG shores and promoters may be sensitive to *Kdm5c* dosage, though the effect is more pronounced with complete loss of KDM5C function.

The *Kdm5d* mutation was associated with both lower and higher methylation at enhancer regions, and DMCs were underrepresented in CpG islands (Fig. 2A). Hypomethylated DMCs were enriched at weak (OR = 5.47, *p =* 4.04 × 10^− 19^) and poised enhancers (OR = 2.74, *p =* 6.76 × 10^− 10^). Hypermethylated DMCs were enriched at strong TSS-proximal enhancers (OR = 3.39, *p =* 0.006) and weak enhancers (OR = 3.26 *p =* 1.36 × 10^− 5^). While both hypo- and hypermethylated DMCs were enriched at weak enhancers, the magnitude of enrichment was greater for hypomethylated DMCs (Fig. 2C).

All mutant contrasts showed enrichment of hypermethylated DMCs in intergenic regions (C+/- vs. C+/+ *p =* 3.17 × 10^− 06^; C- vs. C + *p =* 1.47 × 10^− 18^; D- vs. D + *p =* 1.34 × 10^− 10^) and slight enrichment of hypermethylated DMCs in polycomb-associated chromatin (H3K27me3) (C+/- vs. C+/+ *p =* 0.02; C- vs. C + *p =* 0.001; D- vs. D + *p =* 0.01) (Fig. 2C).

### *Kdm5c* and *Kdm5d* mutations influence DNA methylation at H3K4me1-enriched regions

**Fig. 3:**
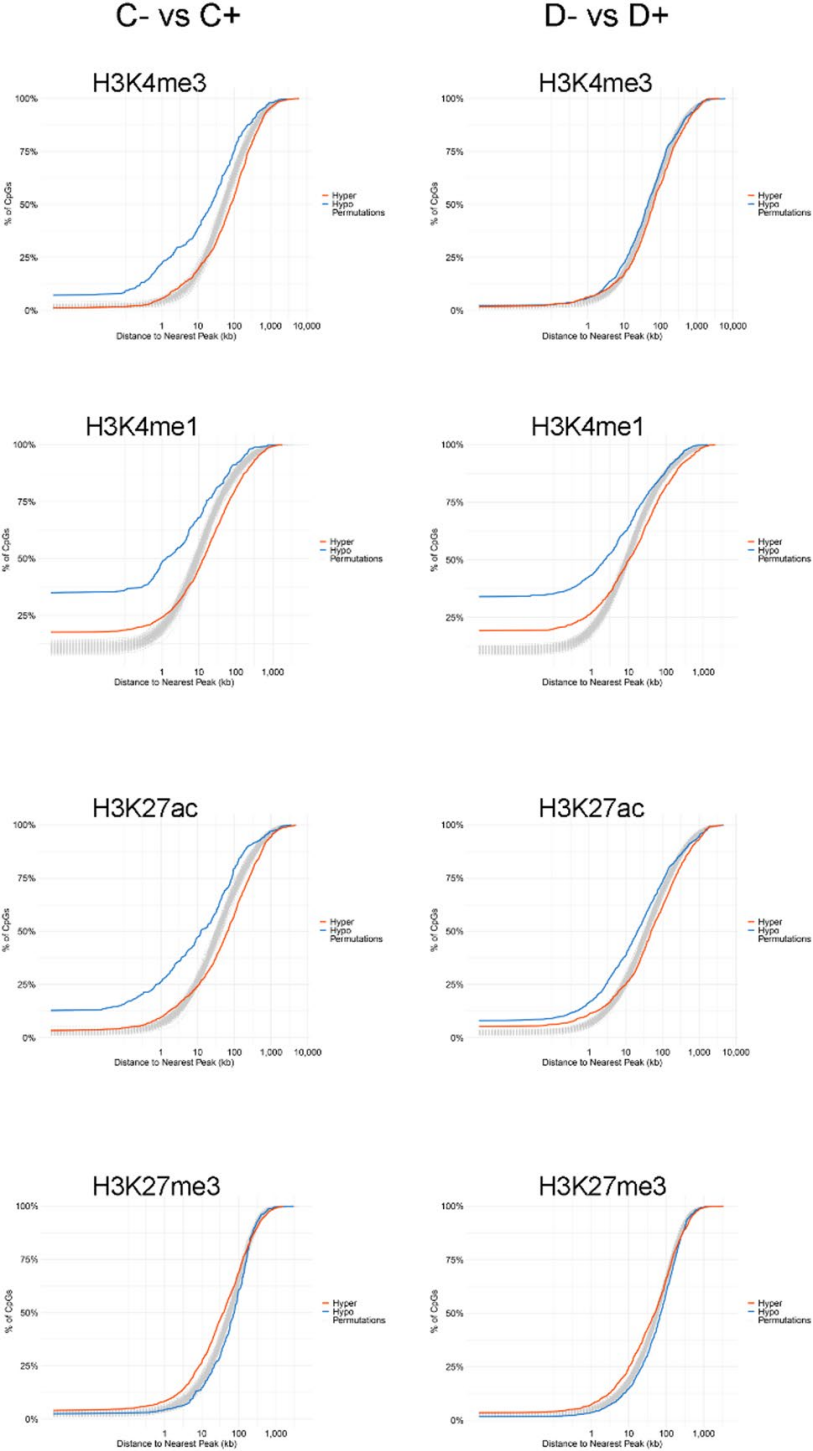
Cumulative distributions of distances between DMCs and nearest histone H3K4me3, H3K4me1, H3K27ac, or H3K27me3 peak

Since our methylation analysis was done in adult livers, next, we used ENCODE data for adult male livers [33] to examine whether the trends observed in P0 livers were also present in adults. ENCODE data for adult liver are limited to male mice, hence, our colocalization tests were performed using DMCs from the mutant male contrasts only. We investigated whether mutant-associated DMCs tended to occur closer to specific chromatin features than expected by chance by analyzing their distances to peaks of H3K4me3 (active promoters), H3K4me1, H3K27ac (active enhancers), and H3K27me3 (polycomb-mediated repression) (Fig. 3). For each DMC, we calculated the distance to the nearest histone mark peak and compared the resulting distribution to a null distribution generated by randomly sampling CpG sites 100 times and recalculating distances.

Hypomethylated DMCs in male C- mutants were located closer to H3K4me3 peaks compared to the null distribution, with approximately 25% mapping within 1 kb of a H3K4me3 peak (Fig. 3). In contrast, hypomethylated DMCs in D- mutants showed a distribution that closely followed the null. Hypomethylated DMCs in both mutant male groups tended to reside near H3K4me1-enriched regions, with ~ 50% of C- vs. C + and ~ 45% of D- vs. D+ DMCs located within 1 kb of a H3K4me1 peak. Both groups also showed a strong tendency for hypomethylated DMCs to be located near H3K27ac peaks, with this pattern more pronounced in the C- mutant contrast compared to the null distribution. Specifically, ~ 25% of C- hypomethylated DMCs were located within 1 kb of an H3K27ac peak, compared to ~ 15% in the D- mutant contrast. Hypermethylated DMCs in both groups were more frequently located near regions marked by the repressive histone modification H3K27me3.

### *Kdm5c* and *Kdm5d* are not major contributors to sex-chromosome complement dependent DNA methylation in mouse liver

To test our hypothesis that *Kdm5c* or *Kdm5d* contributed to sex-chromosome complement-dependent DNA methylation in adult mouse liver, we identified DMRs in the same six contrasts, as described above (Table 1 and Supplementary Tables 1 and 2). As seen with DMCs, the C + vs. D+ contrast detected quite a few DMRs that reflected genetic variation among ICR mice (Supplementary Tables 1 and 2). To minimize the effects of genetic background, DMRs that overlapped with those from the C + vs. D+ contrast were excluded from further analyses. The numbers of DMRs identified in each contrast are listed in Table 1, and all DMRs are provided in Supplementary Table 2. Similar to DMCs, both hypo- and hypermethylated DMRs were detected in contrasts and on all chromosomes (Supplementary Fig. 1). For both sex-biased contrasts (C+/+ vs. C + and C+/- vs. C-), DMRs were enriched on the X chromosome compared to the autosomes (Supplementary Fig. 1C-E). In both contrasts, approximately 70% of the X chromosomal DMRs were hypermethylated in the male groups (C + and C-) (Table 1, Supplementary Fig. 1E). The lower-than-expected number of DMRs hypermethylated in females likely reflects the depletion of X-linked CGIs following stringent filtering, as described in Methods.

We next asked whether *Kdm5c*- and *Kdm5d*-sensitive DMRs overlapped with sex-biased DMRs from wild type mice (C+/+ vs. C+) or those identified in our previous study, which used mice with different combinations of sex-chromosome complement and gonadal sex that permitted separating the effects of the sex chromosomes from those of gonadal sex hormone pathways [3]. The contrast between XX females (XX.F) and sex-reversed XY females (XY.F) detected DMRs that were sensitive to the sex-chromosome complement, whereas the DMRs detected in the contrast between females with monosomy X (XO.F) and XX littermates (XX^*Paf*^.F) were presumably sensitive to X-dosage. If *Kdm5c* or *Kdm5d* were responsible for the effects of X-dosage or the Y chromosome on DNA methylation, respectively, we would expect to see overlaps between mutation-sensitive DMRs and those associated with either X-dosage or the presence of the Y chromosome. Moreover, some of the wild-type sex-biased DMRs would be lost and new DMRs would appear in the C+/- vs. C- contrast.

DMRs from different contrasts were intersected and considered overlapping if they shared at least one nucleotide (Table 2). A total of 35 DMRs were identified as sensitive to *Kdm5c* dosage in the C+/- vs. C+/+ contrast, all of which were located on the autosomes (Table 1 and Supplementary Fig. 1). Contrary to expectations, no overlaps were found between autosomal *Kdm5c*-dosage sensitive and X-dosage dependent DMRs (C+/- vs. C+/+ females and XX^*Paf*^.F vs. XO.F or XX.F vs. XY.F) (Table 2). Similarly, only one overlap was observed between *Kdm5c* dosage-sensitive DMRs and sex-biased DMRs identified by comparing wild-type male and female littermates. These findings suggest that *Kdm5c* dosage is unlikely to be the primary driver of sex chromosome complement-dependent DNA methylation in the adult mouse liver.

**Table 2:** Overlaps between autosomal DMRs from mutant and sex-biased and sex-chromosome complement associated contrasts

Contrast (number of autosomal DMRs)	C+/- vs. C+/+ (35)	C- vs. C+ (93)	D- vs. D+ (89)
C+/+ vs. C+ (184)	1	7	8
*XX.F vs. XY.F (802)	0	1	0
*XX.F vs. XO.F (649)	0	1	1

* Data from Zhuang et al.2020 [3]

In the C- vs. C + and D- vs. D+ contrasts, 7 and 8 autosomal DMRs, respectively, overlapped with sex-biased DMRs from the same genetic background (C+/+ vs. C+). However, there was minimal overlap between DMRs identified from the C- and D- mutant contrasts and those associated with sex-chromosome complement in mice with a C57BL/6J or mixed C57BL/6J x C3H genetic background (XX^*Paf*^.F vs. XO.F and XX.F vs. XY.F) [3] (Table 2).

Mutations in several genes that contribute to sex-biased regulation of transcription lead not only to loss of sex bias in gene expression or methylation but also gain of sex bias in genes or regions that were not biased in wild type samples [16, 37–40]. Comparison of mutant (C+/- vs. C-) and wild type (C+/+ vs. C+) contrasts identified several regions with loss or gain of sex bias in mutants (Supplementary Fig. 3 A and B**)**. Methylation changes in the C- mutants resulted in a gain of sex-bias in the mutants which was not present in the wild-type contrast (Supplementary Fig. 3B), whereas at certain sex-associated DMRs the C-mutation influenced the magnitude of methylation differences between females and males (Supplementary Fig. 3 C).

In general, the C- and D-mutations affected DNA methylation at different loci with only two DMRs that overlapped between the mutant male contrasts (Supplementary Fig. 4 A). Certain DMRs that were hypomethylated in D- mutants had similar methylation levels in wild type and in C- mutant males (Supplementary Fig. 4B), while other DMRs were affected by the C-mutation, but not the D-mutation (Supplementary Figs. 3B and 5).

### DNA methylation signature of *Kdm5c* mutations

It has been suggested that loss of *Kdm5c* was associated with failure to repress germ line genes in the somatic cells of mutation carriers [34, 41]. To explore whether the *Kdm5c* mutation that we used had a similar impact in adult liver, we examined our dataset for DMRs that overlapped with regions that were previously reported as hypomethylated in epiblast-like *Kdm5c*-knock-out cells (EpiLCs) [34]. Since the EpiLC dataset included 1 kb-regions surrounding transcription start sites (TSS) [34], whereas our dataset was depleted for CGIs that tend to reside in promoters, we used relaxed criteria and allowed a maximum distance of 3 kb between DMRs from the two datasets. We identified 12 DMRs from the C- vs. C+ contrast that were within less than 3 kb from those reported in EpiLCs.

**Fig. 4:**
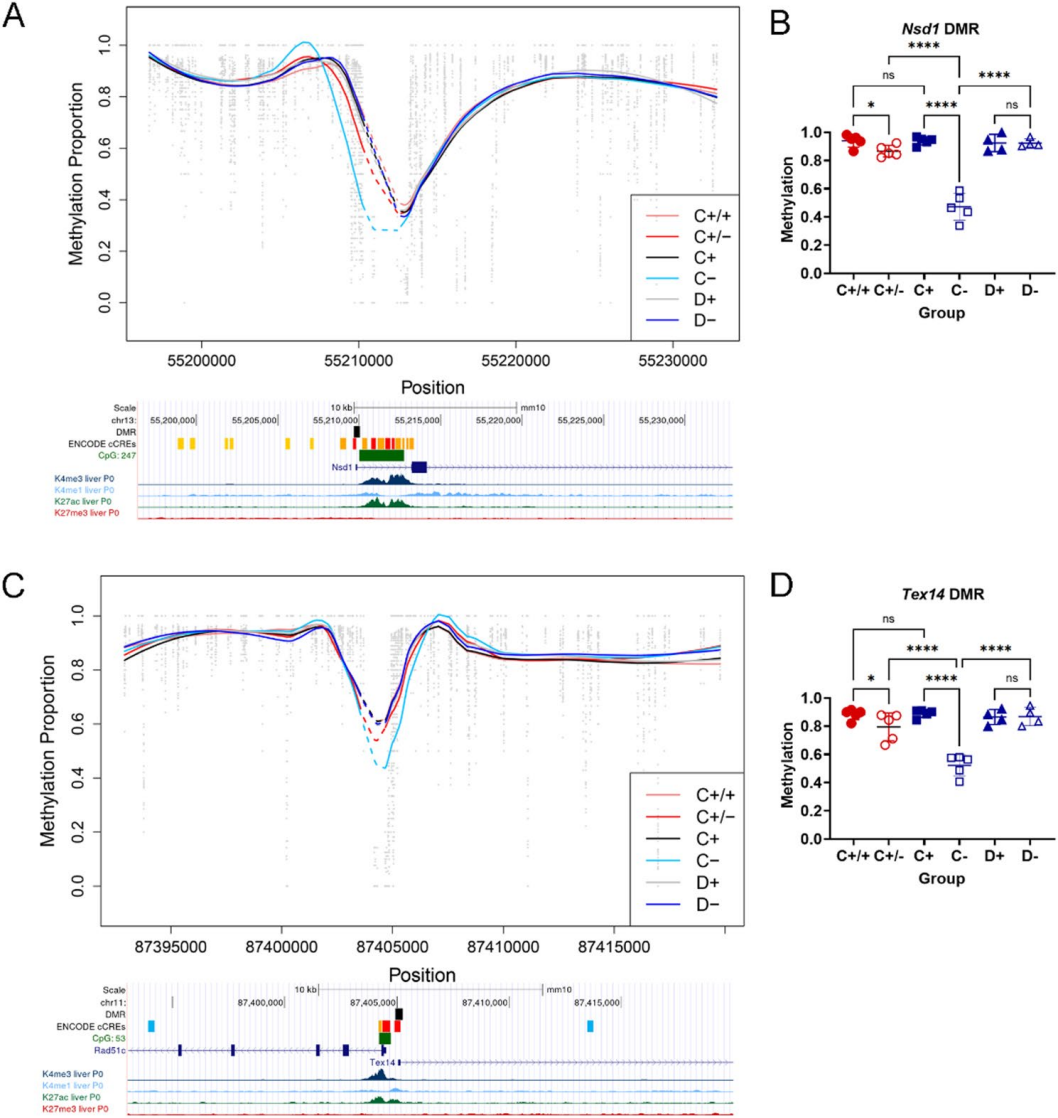
Methylation patterns *Nsd1 *and* Tex14/Rad51c* -associated DMRs overlapping with the EpiLC dataset. (**A**, **C**) Smoothed methylation profiles were generated by expanding the window around the DSS-identified DMR and applying locally weighted regression (LOESS) to estimate methylation trends across CpG sites. These smoothed curves provide an overall view of group-level methylation patterns but may not accurately reflect patterns in regions with sparse data and should not be overinterpreted. Such regions are indicated by dashed lines (**B**, **D**) Methylation plots for the exact DMRs identified by DSS

These DMRs were located near promoters of the following genes: *Gm37912*,* D1Pas1*,* Nr6a1os*,* Gm43697*,* Gm44020*,* Rps4l*,* Zfp469*,* Rad51c*,* Tex14*,* Rnf113a2*,* D430020J02Rik*,* Nsd1*,* Gm6723*,* Usp16*, and *Zfp532* (Fig. 4, Supplementary Figs. 3B and 5). All DMRs had the same direction of methylation change in both datasets, except for *Gm43697*, which was hypomethylated in EpiLCs but hypermethylated in adult liver. It is worth noting that several DMRs were associated with pairs of genes expressed in opposite orientations. Several of these genes are expressed predominantly in germ cells, but some are also highly expressed in adult liver (data from [3, 10], GSE248074) and not sensitive to X-chromosome dosage (*Nsd1*, *Usp16*, *Rps4l*, etc.) (Supplementary Fig. 6). Hence, our data from adult liver are consistent with the data from EpiLCs. This suggests that hypomethylation of these regions is a hallmark of a loss-of-function mutations in *Kdm5c*.

## Discussion

### Sex-biased DNA methylation in mouse liver

Our previous work demonstrated that the sex chromosomes contribute to sex-biased DNA methylation in the mouse liver [3]. To investigate the mechanisms underlying this effect, we hypothesized that sex chromosome-encoded genes, particularly those with sex-specific dosage differences, may mediate sex-biased DNA methylation.

Our analysis showed that the sex-biased contrasts (C+/+ vs. C + and C+/- vs. C-) yielded the highest numbers of DMCs and DMRs, highlighting the strong influence of sex on autosomal and X-linked DNA methylation. To determine whether *Kdm5c* or *Kdm5d* contribute to this sex bias, we examined the overlap between mutation-sensitive regions and sex-biased DMRs. We found modest overlap with 7 and 8 sex-biased regions that were sensitive to *Kdm5c* or *Kdm5d* mutations in males, respectively. One DMR (chr3:59,262,224–59,263,235), shared across all three contrasts, mapped to purinergic receptor P2Y, G-protein coupled 12 (*P2ry12)*, a gene with highest expression in mouse brain and implicated in sexually dimorphic behavioral phenotypes [42].

Several sDMRs that are common between wild type and mutant female vs. male contrasts map to TF-encoding genes that are expressed in a sex biased fashion before puberty and are also pivotal for sex biased regulation in mouse liver (including *Esr1*, *Cux2* and *Onecut1*) (Supplementary Table 2) [3, 16]. This suggests that the loss or lower dosage of *Kdm5c* were unlikely to be responsible for the sex bias in methylation that was previously reported in prepubescent mice [16].

In the *Kdm5c* dosage-sensitive contrast (C+/- vs. C+/+), only one DMR overlapped with a sex-biased region, located at the pentatricopeptide repeat domain 2 (*Ptcd2*) locus, a gene involved in RNA binding and mitochondrial RNA stabilization [43]. The limited overlap suggests that reduced *Kdm5c* dosage alone is insufficient to broadly impact sex-biased methylation and that one functional copy of *Kdm5c* likely compensates the loss of the other copy in heterozygous females.

Minimal to no overlap was observed between mutation-sensitive DMRs and those associated with the sex-chromosome complement, suggesting that *Kdm5c* and *Kdm5d* are unlikely to be the primary mediators of sex chromosome effects on sex-biased DNA methylation in the adult mouse liver.

### Effects of *Kdm5c* and *Kdm5d* mutations

Our data suggest that both *Kdm5c* and *Kdm5d* influence the liver methylome. Male hemizygous mutants (C- vs. C + and D- vs. D+) showed comparable numbers of DMCs and DMRs, whereas female heterozygous mutants (C+/- vs. C+/+) exhibited fewer methylation changes (Table 2 and Supplementary Tables 1 and 2).

In C- mutants, we observed significant enrichment of hypomethylated DMCs at promoters and CpG shores, regions typically marked by H3K4me3. A similar but weaker pattern in C+/- females suggests partial maintenance of normal methylation by the remaining *Kdm5c* allele. In principle, this pattern aligns with the known antagonism between H3K4me3 and DNA methylation, where H3K4me3 prevents the recruitment of DNMT3L, a cofactor that enhances *de novo* DNA methylation by DNMT3A/B [44], and reviewed in [45]. Loss of function of the KDM5C protein in C- males may increase H3K4me3 near promoters, reducing DNA methylation, whereas one functional *Kdm5c* allele in females mitigates this effect. Supporting this, hypomethylated DMCs in C- mutants were significantly closer to H3K4me3 peaks than expected by chance. By contrast, D- mutants lacked such enrichment of hypomethylated DMCs near promoters and H3K4me3-enriched regions, supporting functional divergence between the paralogs. Due to lack of H3K4me2 ENCODE data for adult liver, we are not discussing the impacts of the mutations on DNA methylation in the vicinity of H3K4me2-enriched sites, albeit this mark may be of particular interest for KDM5C- and KDM5D-sensitive regions as it tends to localize downstream of promoters as often do sDMRs [3, 46].

Additionally, hypomethylated DMCs in both C- and D- mutants were closer to enhancer-associated histone marks H3K4me1 and H3K27ac than expected by chance, with a higher proportion of C- DMCs near H3K27ac peaks. Although our data reflect DNA methylation rather than direct chromatin states, these associations suggest that both KDM5C and KDM5D may influence DNA methylation of enhancers.

### Shared targets of *Kdm5c* mutations

Comparative analysis of published data from XY EpiLCs that carry a deletion of exons 11 and 12 of *Kdm5c* [34] and our adult liver dataset revealed DNA methylation changes associated with *Kdm5c* loss. We identified a set of overlapping regions, all with lower methylation levels in mutant livers, and all but one with the same direction of methylation changes in both datasets. It is worth noting that the genetic backgrounds and inclusion criteria for methylation analysis are different in the two datasets, making the overlaps even more suggestive. Together, our findings imply that target loci for KDM5C-dependent methylation are conserved across different cell types and developmental stages and may represent an epigenetic signature of an early impact of KDM5C functional deficiency whose remnants persist throughout development as an epigenetic memory without necessarily affecting expression levels [47, 48].

### Study limitations and mitigating strategies

Hemizygous *Kdm5c* male embryos are not viable on the inbred C57BL/6 background [23] but are viable on F1 or outbred backgrounds [22, 25]. The *Kdm5c* and *Kdm5d* mutations that were used in our study were generated on the outbred ICR strain background [22], resulting in considerable genetic variability within and between the crosses. Genetic variation has a dramatic impact on DNA methylation [49]. The high genetic variability of the ICR mice posed challenges for DNA methylation analysis, particularly in distinguishing the impacts of the induced mutations from those of genetic background variation. To address this, we filtered out CpGs that overlapped with known SNPs, using a comprehensive list of polymorphic sites compiled from SNP calls across 36 inbred mouse strains [50]. We next applied a conservative filter, retaining only cytosines with non-zero coverage across all samples, as zero coverage may reflect unannotated SNPs. This filtering step led to reduced representation of CpG islands in our dataset, which reflects inherent technical challenges. CpG islands are regions with high GC content and often low methylation levels, features that can reduce alignment efficiency and sequencing coverage following sodium bisulfite treatment. Although this stringent filtering reduced the number of CpG sites available for differential methylation analysis, it increased confidence in the accuracy of the data. Consequently, the number of DMRs identified in pairwise contrasts was likely reduced, potentially limiting the detection of overlapping regions in downstream analyses. As a third step to minimize the influence of genetic background, we limited our analysis of mutation effects to contrasts between mice from the same cross.

Another important limitation relates to the method used for differential methylation analysis. Specifically, different tools use distinct strategies for defining DMR boundaries, which can influence results [3]. In Bonefas et al., 2024, DMRs in EpiLCs were identified using methylKit, whereas our analysis used DSS. While methylKit uses logistic regression and incorporates sequencing depth, it does not model biological variability across replicates [51]. In contrast, DSS uses a beta-binomial model that accounts for both technical and biological variation, which is an important consideration given the variability present in our dataset [51]. However, a limitation of beta-binomial approaches, including DSS, is the lack of spatial smoothing between neighboring CpG sites [51]. Therefore, the choice of differential methylation analysis method introduces a potential source of variation that should be considered when interpreting results.

Our data provide a snapshot of the impacts of the mutations on DNA methylation in adult liver and permit identification of distinct targets of the two mutations. They also suggest that sex-chromosome complement dependent methylation in adult liver is unlikely to be due to the influence of KDM5C or KDM5D alone. However, interaction with other factors cannot be ruled out.

## Conclusions

- Mutations in *Kdm5c* and *Kdm5d* induce significant changes in DNA methylation in adult mouse liver, primarily at autosomal loci.
- Functional annotation indicates that loss of the KDM5C function affects DNA methylation at promoter and enhancer regions enriched for histone H3K4me3 and H3K4me1 marks.
- Overall, *Kdm5c* and *Kdm5d* are not the primary mediators of sex-chromosome complement effects on DNA methylation in the adult liver.

## Supplementary Information

Supplementary Material 1.

Supplementary Material 2.

## Data Availability

All data generated or analysed during this study are included in this published article and its supplementary information files. Sequencing data have been deposited to NCBI (GSE307211).
