# Supplementary material for: KDM5C and KDM5D influence DNA methylation in adult mouse liver
Source: Biol Sex Differ. 2026 Feb 12;17:55. doi: 10.1186/s13293-026-00844-6 (PMC13011546; doi:10.1186/s13293-026-00844-6)
Supplement: Supplementary file 1 — Supplementary Material 1. [file 13293_2026_844_MOESM1_ESM.pdf]

Supplementary information for

KDM5C and KDM5D influence DNA methylation in adult mouse liver

Emily Gibbons[1], Kathleen Oros Klein[2], Shinya Inoue[3], Tohru Kimura[3], Celia M. T. Greenwood[2,4], Anna K. Naumova[1,5,6]

1.Department of Human Genetics, McGill University, Montréal, Québec, Canada
2. Lady Davis Institute for Medical Research, Montreal, Québec, Canada
3. Laboratory of Stem Cell Biology, Department of Biosciences, Kitasato University School of Science, 1-15-1 Kitasato, Minami-ku, Sagamihara, Kanagawa, Japan
4. Gerald Bronfman Department of Oncology, and the Department of Epidemiology, Biostatistics & Occupational Health, McGill University, Montréal, Québec, Canada
5.The Research Institute of the McGill University Health Centre, Montréal, Québec, Canada
6. Department of Obstetrics and Gynecology, McGill University, Montréal, Québec, Canada

Supplementary Table 1. Number of DMRs and DMCs prior to C+ vs D+ overlap removal

Contrast	Autosomes		X chromosome		Y chromosome	
	DMCs (hypo vs hypermethylated)	DMRs (hypo vs hypermethylated)	DMCs (hypo vs hypermethylated)	DMRs (hypo vs hypermethylated)	DMCs (hypo vs hypermethylated)	DMRs (hypo vs hypermethylated)
C+/- vs C+/+	1219 (314 vs 905)	38 (10 vs 28)	25 (6 vs 19)	0	-	-
C- vs C+	2288 (550 vs 1738)	102 (42 vs 60)	61 (13 vs 48)	2 (1 vs 1)	6 (1 vs 5)	0
D- vs D+	2925 (1578 vs 1347)	119 (76 vs 43)	90 (47 vs 43)	3 (2 vs 1)	5 (1 vs 4)	0
C+ vs D+	3112 (2130 vs 982)	128 (77 vs 51)	104 (84 vs 20)	3 (2 vs 1)	5 (5 vs 0)	0
C+/+ vs C+	3794 (847 vs 2947)	192 (39 vs 153)	3302 (2273 vs 1029)	513 (355 vs 158)	-	-
C+/- vs C-	3901 (982 vs 2919)	191 (25vs 166)	3274 (2334 vs 940)	459 (317 vs 142)	-	-

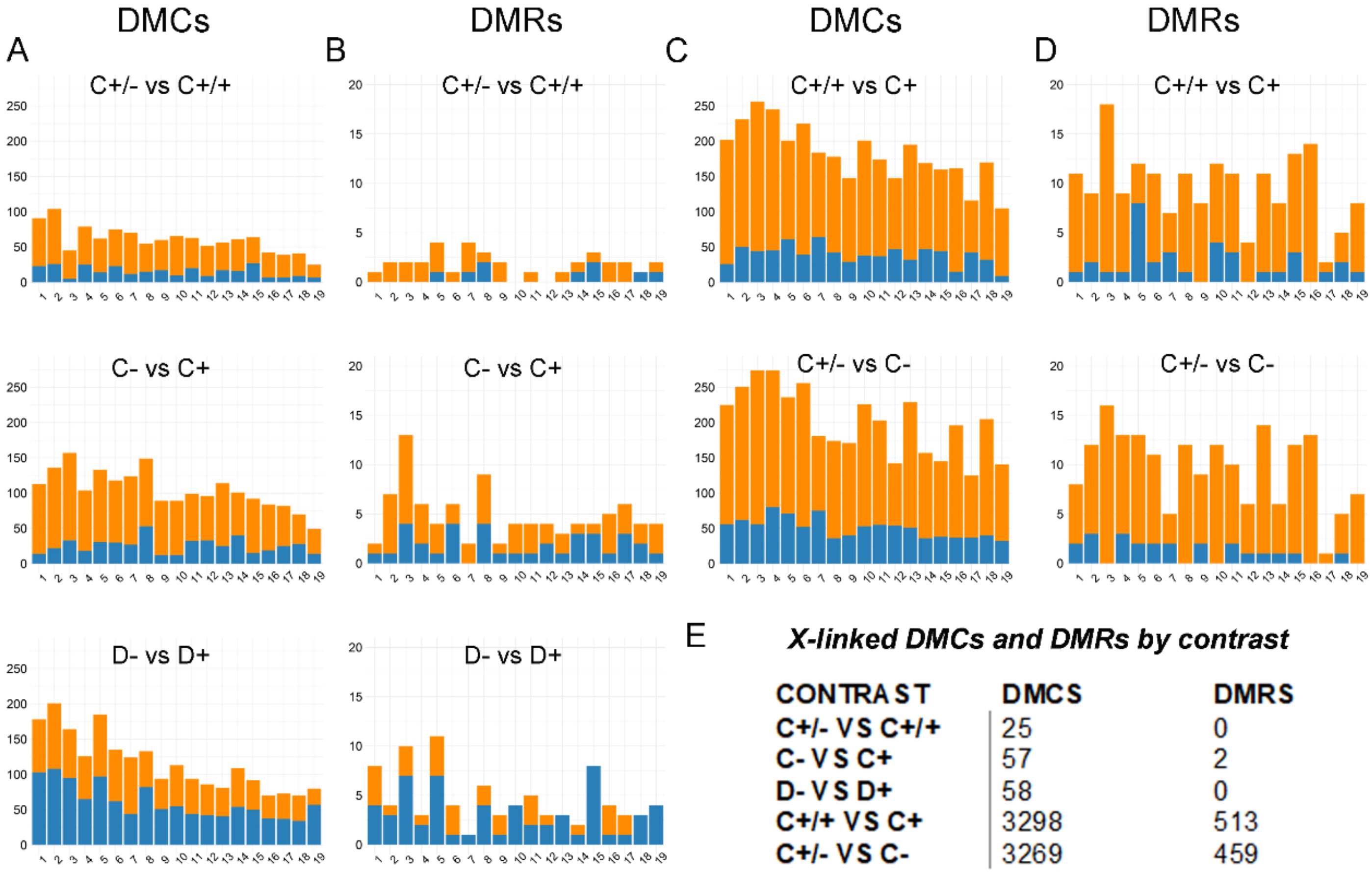

Supplementary Fig. 1. Distribution of DMCs and DMRs identified using DSS across all contrasts. Bar plots show autosomal DMCs for mutant contrasts (A) and sex-biased contrasts (C), and autosomal DMRs for mutant contrasts (B) and sex-biased contrasts (D). For each contrast, bars are divided by methylation direction: regions hypermethylated in the first group listed (i.e., the group on the left of the "vs" sign) are shown in orange, and those hypermethylated in the second group (i.e., the group on the right of the “vs” sign) are shown in blue. X chromosome DMCs and DMRs are summarized separately in panel (E).

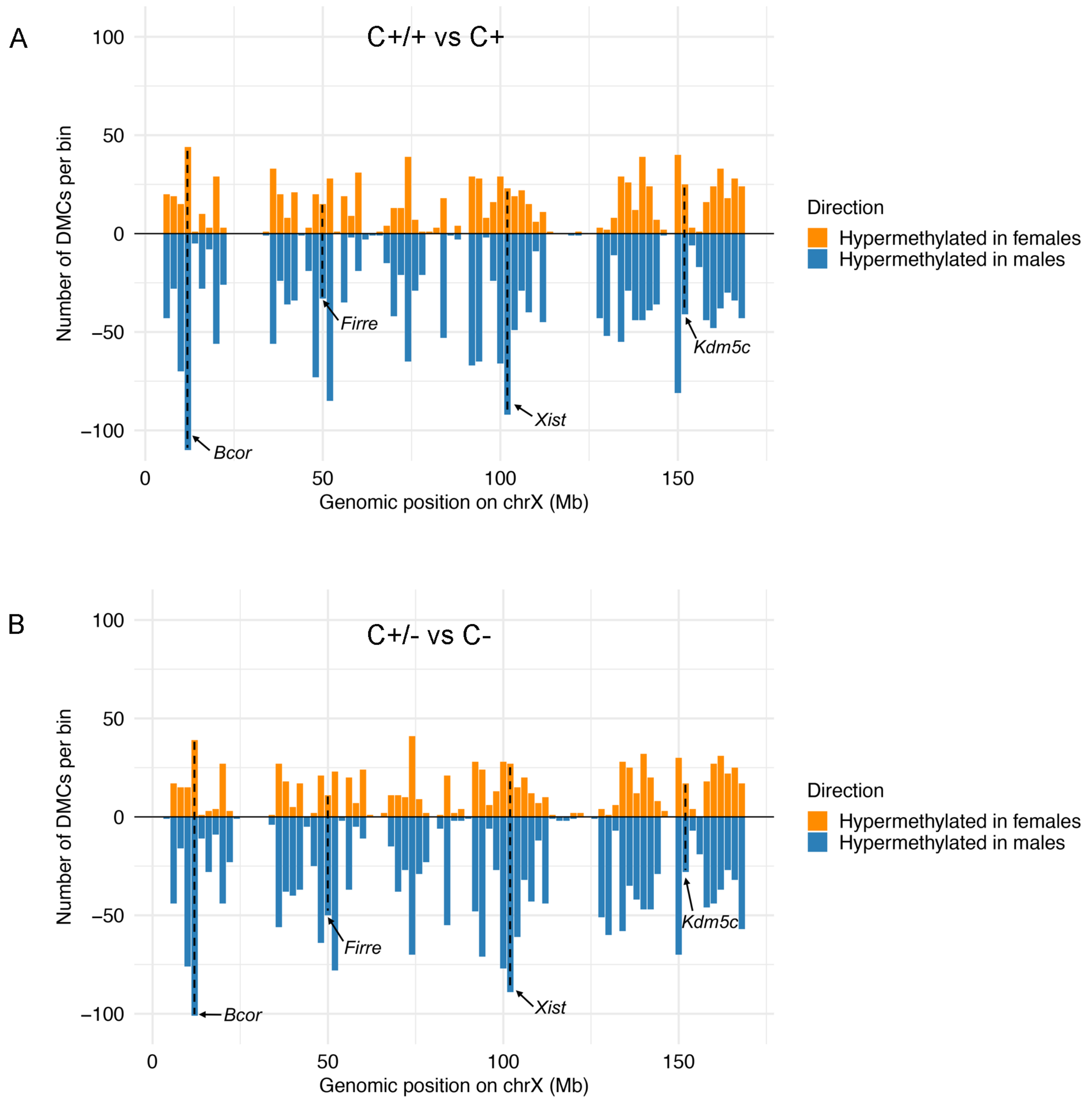

Supplementary Fig. 2. Distribution of sDMCs along the X chromosome. (A) The wild-type sex-biased contrast (C+/+ vs C+) and (B) the mutant sex-biased contrast (C+/- vs C-). DMCs were grouped into 2 Mb bins along the X chromosome. Bars represent the number of DMCs per bin, with positive values indicating loci hypermethylated in females (orange) and negative values indicating loci hypermethylated in males (blue). Bins containing example loci (e.g., *Bcor, Firre, Xist, Kdm5c*) are indicated by arrows.

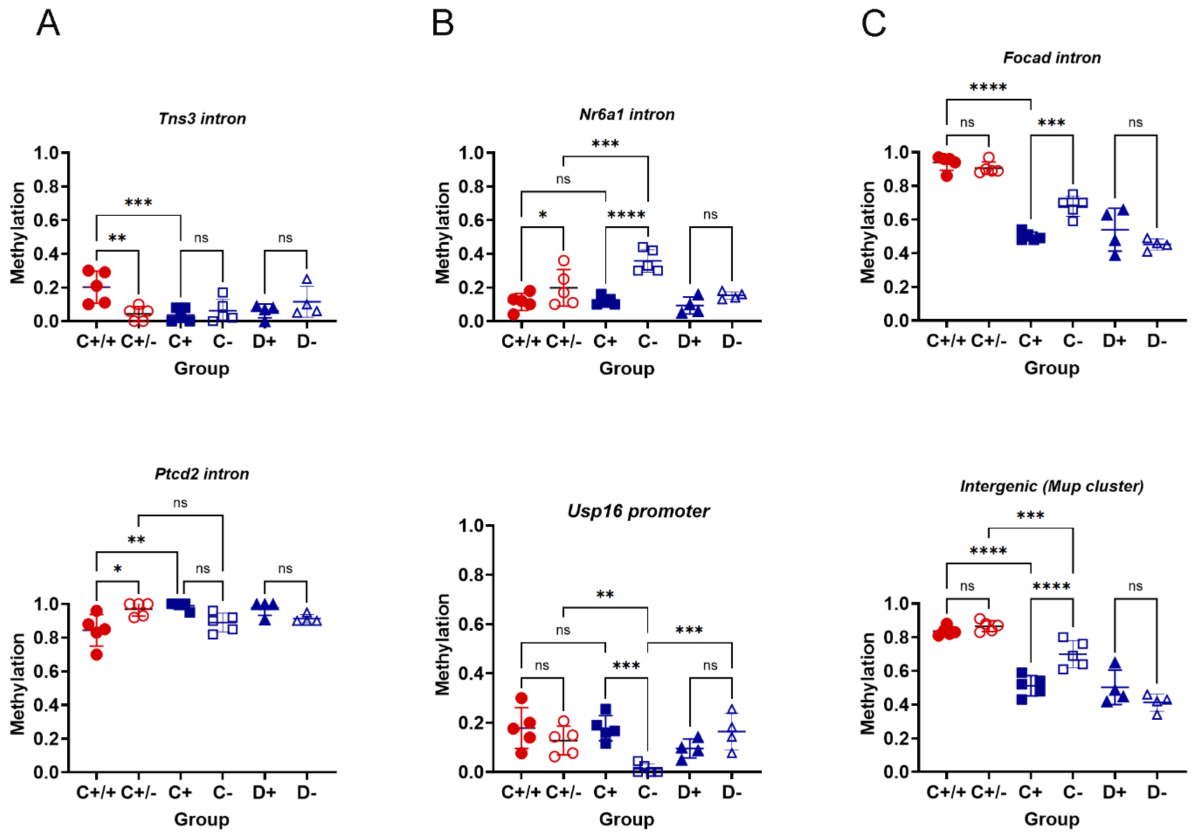

Supplementary Fig. 3. Examples of effects of *Kdm5c* mutations on sex-biased DNA methylation in adult mouse liver. (A) Examples of loss of sex bias in methylation in the mutant female vs male contrasts. (B) Examples of gain of sex bias in methylation in the mutant female vs male contrasts. (C) Examples of change in the magnitude of sex bias in methylation in the mutant female vs male contrasts compared to wild type females and males. Asterisks show *p*-values: * *p*<0.05, ** *p*<0.01, *** *p*<0.001, *** *p*<0.0001 (Fisher's LSD test), ns - non-significant.

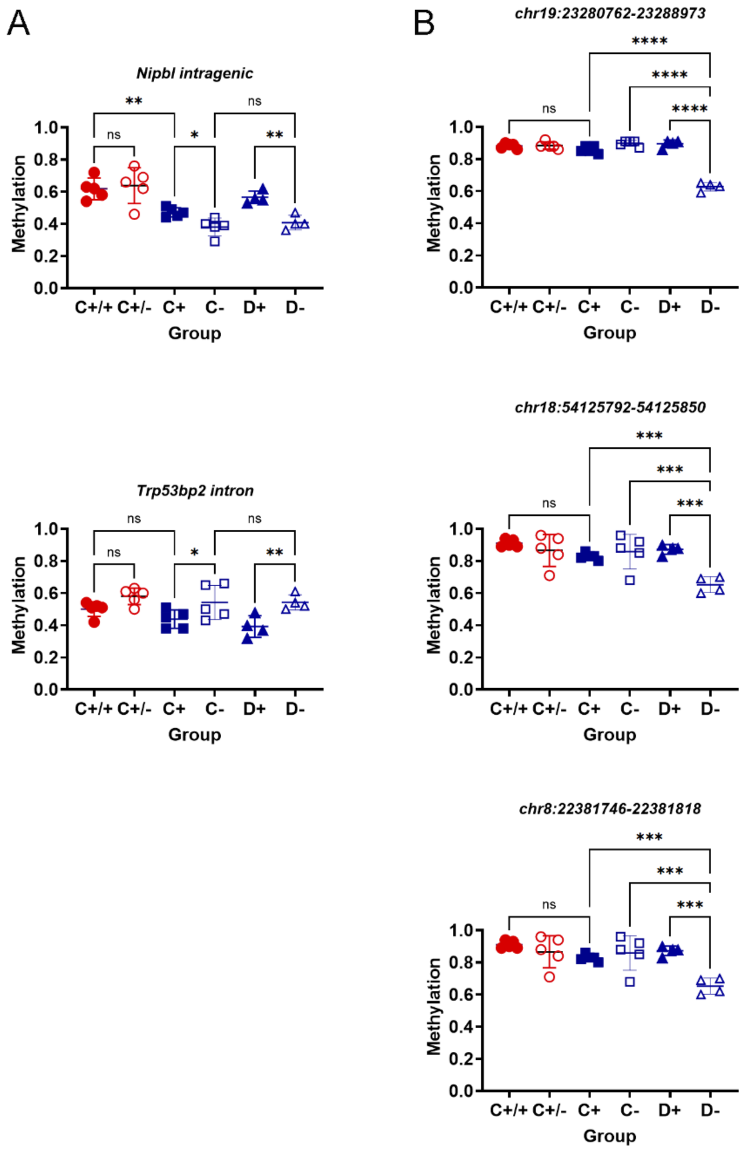

Supplementary Fig. 4. Examples of effects of the *Kdm5d* mutation on DNA methylation in adult mouse liver. (A) Loci where C- and D- mutants show similar methylation changes relative to WT groups. At the *Nipbl* region, both male mutants lose DNA methylation, whereas at the *Trp53bp2* region, both gain DNA methylation. (B) Examples of DMRs where D- mutants exhibit a loss of DNA methylation. Asterisks show *p*-values: * *p*<0.05, ** *p*<0.01, *** *p*<0.001, *** *p*<0.0001 (Fisher's LSD test), ns - non-significant.

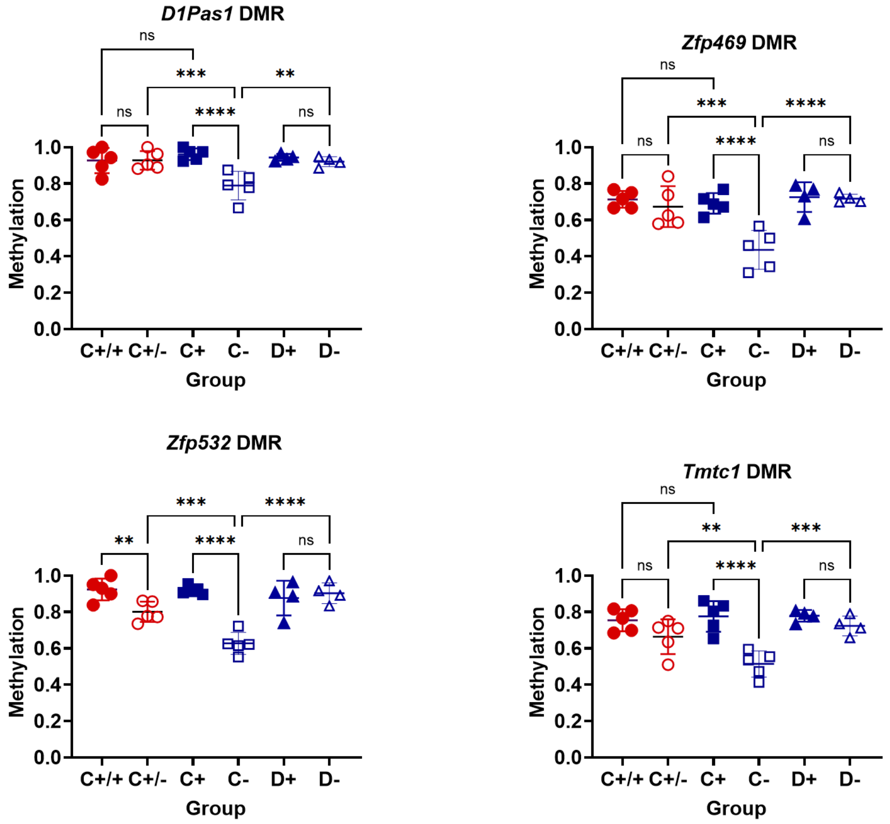

Supplementary Fig. 5. Examples of methylation data for DMRs that are common between C-mutant adult liver and knock-out EpiLCs (34). *D1Pas1* and *Zfp532* DMRs map to the promoters of these genes. The *Zfp469* and *Rsp4l* DMRs map to intragenic regions but overlap with H3K4me3 peaks, based on ENCODE data for P0 liver (UCSC genome browser). Asterisks show *p*-values: * $p<0.05$, ** $p<0.01$, *** $p<0.001$, *** $p<0.0001$ (Fisher's LSD test), ns - non-significant.

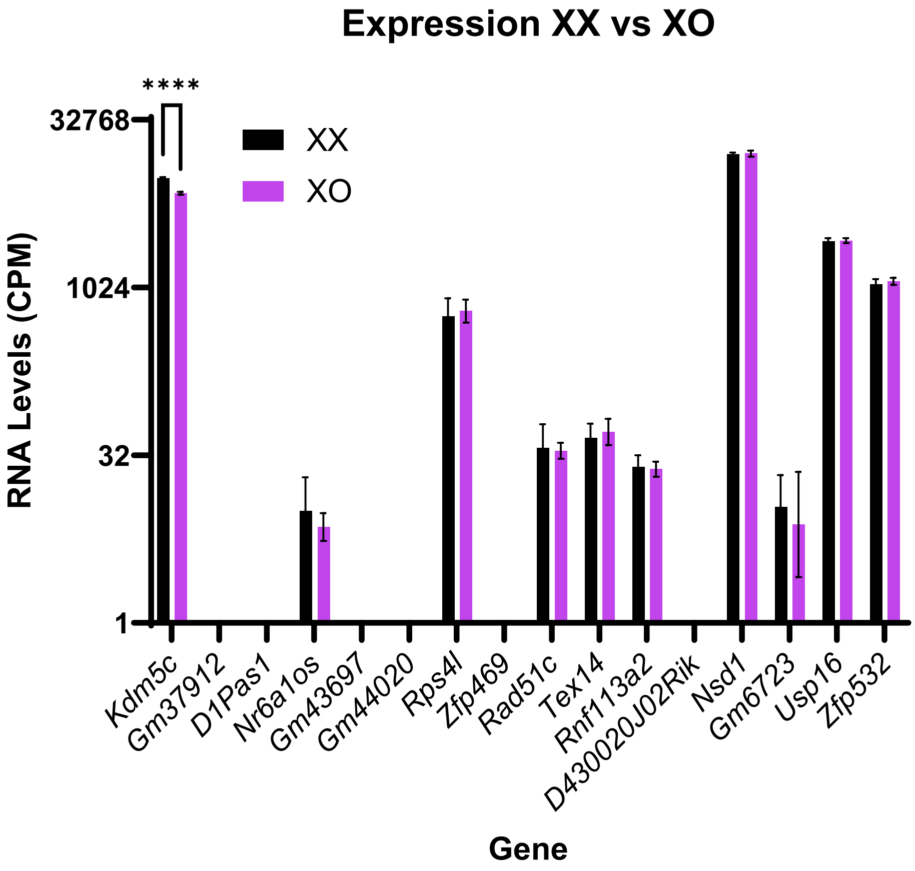

Supplementary Fig. 6. **Expression levels of *Kdm5c* and genes associated with C- vs C+ DMRs in adult mouse livers of XX*Paf*.F and XO.F.** Y-axis shows RNA levels using Log2 scale. Significant difference in expression levels is indicated by asterisks: **** p< 0.001. Data from GSE248074 (3, 10).
